# The role of thromboxane prostanoid receptor signaling in gastric ulcer healing

**DOI:** 10.1111/iep.12410

**Published:** 2021-10-16

**Authors:** Sakiko Yamane, Hideki Amano, Yoshiya Ito, Tomohiro Betto, Yoshio Matsui, Wasaburo Koizumi, Shuh Narumiya, Masataka Majima

**Affiliations:** ^1^ Department of Pharmacology Thoracic Surgery Kitasato University School of Medicine Kanagawa Japan; ^2^ Department of Gastroenterology Thoracic Surgery Kitasato University School of Medicine Kanagawa Japan; ^3^ Department of Gastroenterology Drug Discovery Medicine Kyoto University Graduate School of Medicine Kyoto Japan

**Keywords:** angiogenesis, gastric ulcer healing, TGF‐β, TXA_2_, VEGF‐A

## Abstract

The process of gastric ulcer healing includes cell migration, proliferation, angiogenesis and re‐epithelialization. Platelets contain angiogenesis stimulating factors that induce angiogenesis. Thromboxane A_2_ (TXA_2_) not only induces platelet activity but also angiogenesis. This study investigated the role of TXA_2_ in gastric ulcer healing using TXA_2_ receptor knockout (TPKO) mice. Gastric ulcer healing was suppressed by treatment with the TXA_2_ synthase inhibitor OKY‐046 and the TXA_2_ receptor antagonist S‐1452 compared with vehicle‐treated mice. TPKO showed delayed gastric ulcer healing compared with wild‐type mice (WT). The number of microvessels and CD31 expression were lower in TPKO than in WT mice, and TPKO suppressed the expression of transforming growth factor beta (TGF‐β) and vascular endothelial growth factor A (VEGF‐A) in areas around gastric ulcers. Immunofluorescence assays showed that TGF‐β and VEGF‐A co‐localized with platelets. Gastric ulcer healing was significantly reduced in WT mice transplanted with TPKO compared with WT bone marrow. These results suggested that TP signalling on platelets facilitates gastric ulcer healing through TGF‐β and VEGF‐A.

## 1 INTRODUCTION

The process of ulcer healing is complex, including the formation of granulation tissue, contraction of the ulcerated tissue, angiogenesis and re‐epithelialization.^1^, ^2^ Angiogenesis, defined as the formation of new blood vessels by the extension or elaboration of existing vasculature, is controlled by various angiogenesis stimulating factors, such as vascular endothelial growth factor (VEGF) and transforming growth factor (TGF)‐β.^3^, ^4^

Platelets play an important role in homeostasis, tissue repair, maintenance of endothelium, and vascular tone. They may also facilitate the delivery of angiogenesis regulators and other growth factors to sites of pathologic angiogenesis, such as tumour growth and metastasis formation.^5^, ^6^ Activation of platelets has been reported to stimulate angiogenesis. Once activated, platelets aggregate, and platelet aggregation is reported to be the cardinal feature of vascular repair.^7^, ^8^ These findings suggested platelet aggregation is key to the process of gastric ulcer healing.

Thromboxane A_2_ (TXA_2_) is generated from arachidonic acid by the sequential action of cyclooxygenase (COX) and TXA_2_ synthase (TXS). Thromboxane A_2_ binds to thromboxane prostanoid (TP) receptor. Thromboxane A_2_ is a potent stimulator of platelet aggregation and smooth muscle constriction and has been associated with pathological conditions such as tumour growth and asthma.^9^, ^10^ Furthermore, TXS/TXA_2_‐TP signalling was shown to induce blood flow recovery by activating platelets, suggesting that TXA_2_ may participate in gastric mucosa injury by inducing platelet aggregation, stimulating the production of angiogenesis stimulating factors.^11^ To date, however, the role of TP signalling in platelet aggregation associated gastric ulcer healing has not been determined. The present study therefore examined whether TXA_2_ enhances gastric ulcer healing by treatment with the TXA_2_ synthase inhibitor OKY‐046 and the TXA_2_ receptor antagonist S‐1452. Additionally, this study evaluated the mechanism by which TP signalling facilitates gastric ulcer healing through the aggregation of platelets that secrete VEGF and transforming growth factor beta (TGF‐β). The results of this study indicate that TP signalling is required for angiogenesis and ulcer healing by VEGF‐A and TGF‐β secreted by platelets.

## 2 Ι MATERIALS AND METHODS

### 2.1 Ethical approval

All animal experimental protocols and procedures were approved by the guidelines for animal experiments of Kitasato University School of Medicine. The experimental procedures followed the United States National Institutes of Health Guide for the Care and Use of Laboratory Animals (NIH publication no. 85‐23, revised 1996).

### 2.2 Animals

Eight‐week‐old male C57Bl/6 N mice were obtained from Crea Japan (Tokyo, Japan). TXA_2_ receptor knockout (TPKO) mice with a C57Bl/6 hybrid background were developed in our laboratory.^12^ Mice were maintained at constant humidity (60 ± 5%) and temperature (25 ± 1°C) on a 12‐h light/dark cycle. All animals were provided food and water ad libitum.

### 2.3 Induction of gastric ulcers and drugs

Mice were anaesthetized by intraperitoneal (i.p.) injection of mixed anaesthetic agents (medetomidine 0.75 mg/kg, midazolam 4 mg/kg and butorphanol 5 mg/kg) and awakened by i.p. injection of atipamezole (0.75 mg/kg). Gastric ulcers were induced by the serosal application of 100% acetic acid, as reported previously.^13^, ^14^, ^15^ The serosal area exposed to 100% acetic acid measured 33.2 mm^2^. The animals were fed normally thereafter. Mice were treated with TP antagonist (S‐1452, 10 mg/kg, orally, Shionogi Co. Ltd.), TXS inhibitor OKY‐046 (50 mg/kg, Kissei Pharmaceutical Co. Ltd.) or vehicle once daily by tube administration, beginning just before ulcer induction and for a total of 7 days. Following ulcer induction, the mice were administered P‐selectin antibody (RB40.34, rat IgG1, 30 μg/mouse, BD Pharmingen) i.p. once daily.

### 2.4 Measurement of ulcerated area

Stomachs were removed surgically and opened along the greater curvature. The ulcer area was measured using Image J software.^13^, ^14^

### 2.5 Immunohistochemical staining

Tissue samples encompassing the gastric ulcer area were immediately fixed in 10% neutral buffered paraformaldehyde, dehydrated with a graded series of ethanol solutions, and embedded in paraffin. The paraffin‐embedded blocks were sliced into 3‐μm sections, and each section was mounted onto a glass slide and stained with haematoxylin and eosin stain (H&E) or processed for immunohistochemistry. For the latter, sections were activated using Histo VT One (Nacalai Tesque) or 0.01 M citrate buffer (pH 6), preincubated with Protein Block Serum‐Free (Dako, Glostrup) and incubated overnight at 4°C with rabbit polyclonal anti‐CD31 antibody (1:200, cat. no. ab28364, Abcam).

Following treatment with primary antibodies, the samples were immersed in 3% hydrogen peroxide (H_2_O_2_) for 30 min, incubated for 30 min at room temperature with N‐Histofine Simple Stain Mouse MAX PO(R) (Nichirei Bioscience), and immersed in 3, 3′‐diaminobenzine. The sections were counterstained with Mayer’s haematoxylin for 30 s and dehydrated, cleared and mounted.

### 2.6 Microvessel density

Microvessel density (MVD) in the ulcer tissues was assessed as a parameter of angiogenesis in a blinded manner, according to previously described methods with some modifications.^13^ For quantification, the numbers of CD31^+^ cells were counted in each animal. Briefly, four to six areas of highest neovascularization were identified by scanning the ulcer area at ×40 and ×100 magnifications. Individual microvessels in these areas were counted on a ×400 field.

### 2.7 Quantitative real‐time PCR analysis

Transcripts encoding TP, TXS, TGF‐β, VEGF‐A and glyceraldehyde 3‐phosphate dehydrogenase (GAPDH) were quantified by real‐time polymerase chain reaction (PCR) analysis. We extracted total RNA from the edge of gastric ulcer granulation tissues with TRIzol reagent (Gibco‐BRL; Life Technologies), and single‐stranded cDNA was generated from 0.5 μg total RNA by reverse transcription with ReverTra Ace (Toyobo). Quantitative PCR was performed using SYBR Premix Ex Taq II (Takara Bio). The DNA sequences of the real‐time PCR primers, designed using Primer 3 software (http://primer3.sourceforge.net/) based on data from GenBank, are described in Table 1. Data were normalized to the level of GAPDH mRNA in each sample.

**TABLE 1:** Mouse gene primers for real‐time PCR

	Sense	Antisense
TP	5´‐GGCTGCTGGCTTCTAAGTGTG‐3´	5´‐TTCCGTGACCGGTAAGTATTG‐3´
TXS	5´‐GGATTCTGCCCAATAAGAACC‐3´	5´‐GAAGTCTCTCCGCCTCTCTTC‐3´
TGF‐β	5´‐AACAATTCCTGGCGTTACCTT‐3´	5´‐TGTATTCCGTCTCCTTGGTTC‐3´
VEGF‐A	5´‐GAGAGAGGCCGAAGTCCTTT‐3´	5´‐TTGGAAC‐CGGCATCTTTATC‐3´
GAPDH	5´‐ACATCAAGAAGGTGGTGAAGC‐3´	5´‐AAGGTGGAAGAGTGGGAGTTG‐3´

### 2.8 Measurement of platelet counts and bleeding time

The number of platelets was counted with a Celltac automatic cell counter (MEK‐6450, Nihon Kohden). Bleeding time was measured by cutting off a 3‐mm segment from the distal end of the tail of each mouse. The amputated tails were maintained horizontally in contact with air at constant temperature. Blood was gently blotted every 15 s with a piece of filter paper, with care taken not to apply pressure to the tip of the tail. The bleeding time was defined as the interval from cutting until no blood appeared on the filter two consecutive times.^16^

### 2.9 Immunofluorescence staining

Tissue samples encompassing the gastric ulcer area were immediately fixed in 10% neutral buffered paraformaldehyde, dehydrated with a graded series of ethanol solutions, and embedded in paraffin. The paraffin‐embedded blocks were sliced into 3‐μm sections, and each section was mounted onto a glass slide. The sections were activated using 0.01 M citrate buffer (pH 6), preincubated with Protein Block Serum‐Free (Dako), and incubated overnight at 4°C with (a) primary anti‐CD41 antibody (1:50, rat monoclonal, sc‐19963, Santa Cruz Biotechnology), (b) primary anti‐TGF‐β1 antibody (1:200, rabbit polyclonal, ab92486, Abcam) and/or (c) primary anti‐VEGFA antibody (1:50, rabbit polyclonal, ab46154, Abcam), and washed three times with phosphate‐buffered saline (PBS). Sections that had been incubated with anti‐CD41 and anti‐TGF‐β1 or anti‐CD41 and anti‐VEGFA antibody were incubated for 1 h at room temperature with Alexa Fluor 488‐conjugated donkey anti‐rat IgG (1:500, Molecular Probes) and Alexa Fluor 594‐conjugated donkey anti‐rabbit IgG (1:500, Molecular Probes). The samples were imaged with an optical photomicroscope BX53/DP72 (Olympus, Tokyo, Japan) and a fluorescence microscope (Biozero BZ‐9000 Series; Keyence).

### 2.10 Bone marrow transplantation

Donor bone marrow (BM) was obtained by flushing the femoral and tibial cavities of WT and TPKO mice with PBS. The flushed BM cells were dispersed and re‐suspended in PBS at a density of 1 × 10^6^ cells/100 μl.^17^ WT and TPKO mice were lethally irradiated with 9.5 Gy X‐rays using an MBR‐1505 R X‐ray irradiator (Hitachi Medico) equipped with a filter (copper, 0.5 mm; aluminium, 2 mm). The cumulative radiation dose was monitored. BM mononuclear cells from WT and TPKO mice (2 × 10^6^ cells/200 μl) were transplanted into irradiated WT and TPKO mice via the tail vein.

### 2.11 Statistical analysis

Data were expressed as mean ± standard deviation (SD). Data in two groups were compared using Student’s *t*‐tests, and data in more than two groups were compared by one‐way analysis of variance (ANOVA) followed by Bonferroni’s post hoc test. All statistical analyses were performed using GraphPad Prism software, version 6 (GraphPad Software). *p* < .05 was considered statistically significant.

## 3 RESULTS

### 3.1 Temporal changes in ulcerated areas and expression of TP and TXS in granulation tissues of gastric ulcers

**FIGURE 1:**
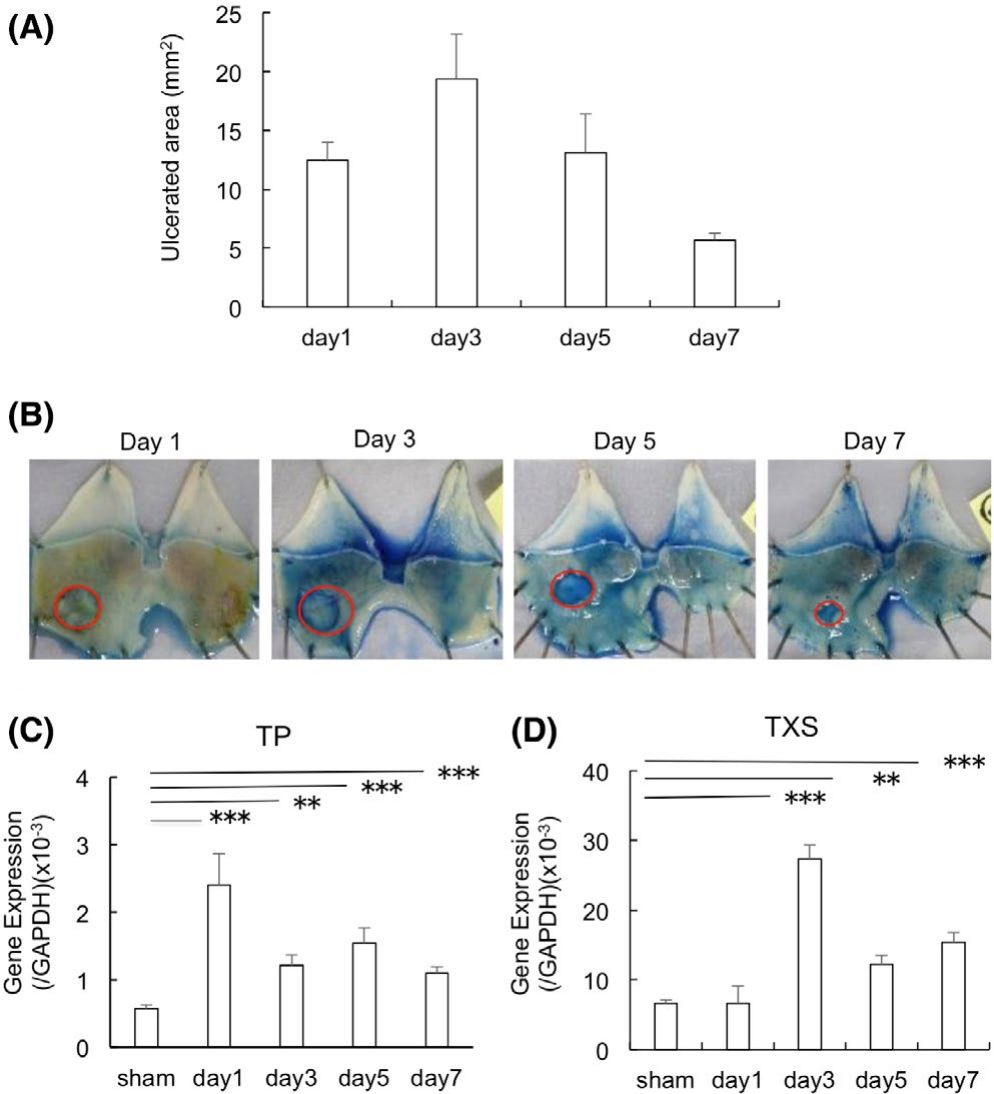
Association of TXS and thromboxane prostanoid (TP) expression with the process of gastric ulcer healing. (A) Time course of ulcer area size following ulcer induction. Error bars indicate the mean ± SD. (B) Typical appearance of gastric ulcer healing over time. (C, D) Time course of levels of TXS mRNA (C) and TP mRNA (D) in gastric ulcer areas. Error bars indicate the mean ± SD. *p < .05 versus vehicle by one‐way ANOVA

Gastric ulcers developed over time after exposure of gastric serosa in WT mice to 100% acetic acid. Average ulcer areas are shown in Figure 1A, and the typical appearances of ulcers on days 1, 3, 5 and 7 are shown in Figure 1B. Ulcerated areas peaked on day 3 and gradually decreased thereafter. To determine whether TP signalling is involved in the natural course of these ulcers, the expression of TP and TXS in gastric ulcer granulation tissues of WT mice was evaluated by real‐time PCR (Figure 1C and D). The levels of TP and TXS mRNAs were higher in mice exposed to 100% acetic acid than in naive mice, suggesting that TXA_2_‐TP signalling is involved in gastric ulcer healing.

### 3.2 Effect of TXA_2_‐TP signalling in gastric ulcer healing

**FIGURE 2:**
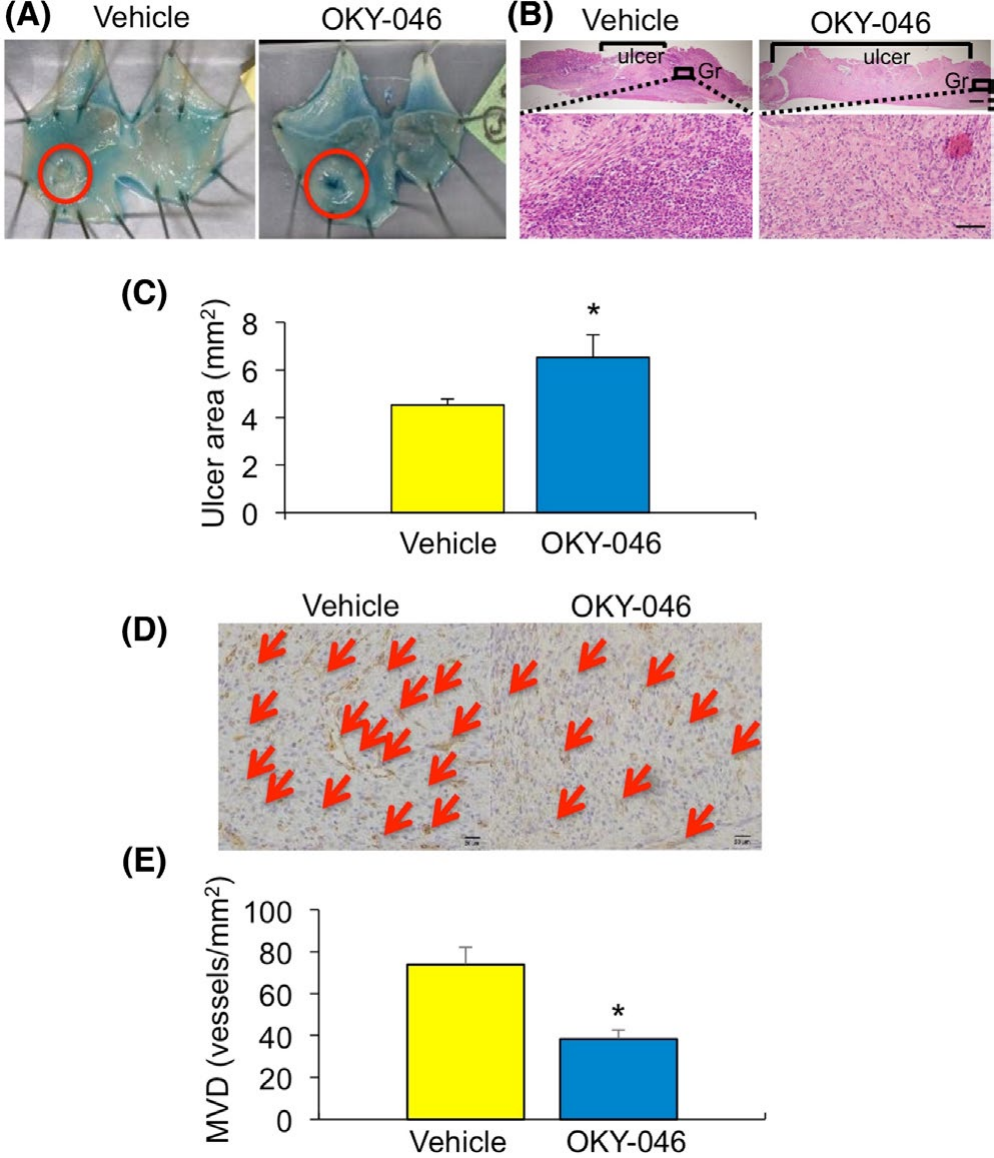
Effect of TXS on healing of acid‐induced gastric ulcers. (A) Typical appearance of ulcers in OKY‐046‐ and vehicle‐treated mice. (B) Haematoxylin–eosin staining in gastric ulcer lesions in OKY‐046‐ and vehicle‐treated mice. Gr, granulation tissue, Bar = 200 μm (upper), Bar = 50 μm (lower) (C) Ulcer areas on day 7 in OKY‐046‐ and vehicle‐treated mice. Error bars indicate the mean ± SD. *p < .05 versus vehicle by Student’s *t*‐test. (D) Representative immunohistochemical staining of CD31 in gastric ulcer lesions from OKY‐046‐ and vehicle‐treated mice. Bar = 20 μm. (E) Microvessel density in ulcer granulation areas of OKY‐046‐ and vehicle‐treated mice on day 7. Error bars indicate the mean ± SD. *p < .05 versus vehicle by Student’s *t*‐test

To examine the effects of TXS on gastric ulcer healing, the effects of the selective TXA_2_ synthase inhibitor OKY‐046 on average ulcer areas were measured. Ulcerated areas on day 7 were significantly larger in mice treated with OKY‐046 than with vehicle (6.54 ± 0.93 versus 4.54 ± 0.24 mm^2^, *p* < .05, *n* = 3–6 per group; Figure 2A,B,C). Immunohistochemical analysis using anti‐CD31 antibody showed that MVD in the ulcer granulation area was significantly lower in mice treated with OKY‐046 than with vehicle (38.43 ± 4.10 versus 74.0 ± 8.16 vessels/mm^2^, *p* < .05, *n* = 3–6 per group; Figure 2D,E). These results suggested that TXS induced ulcer healing in mice.

**FIGURE 3:**
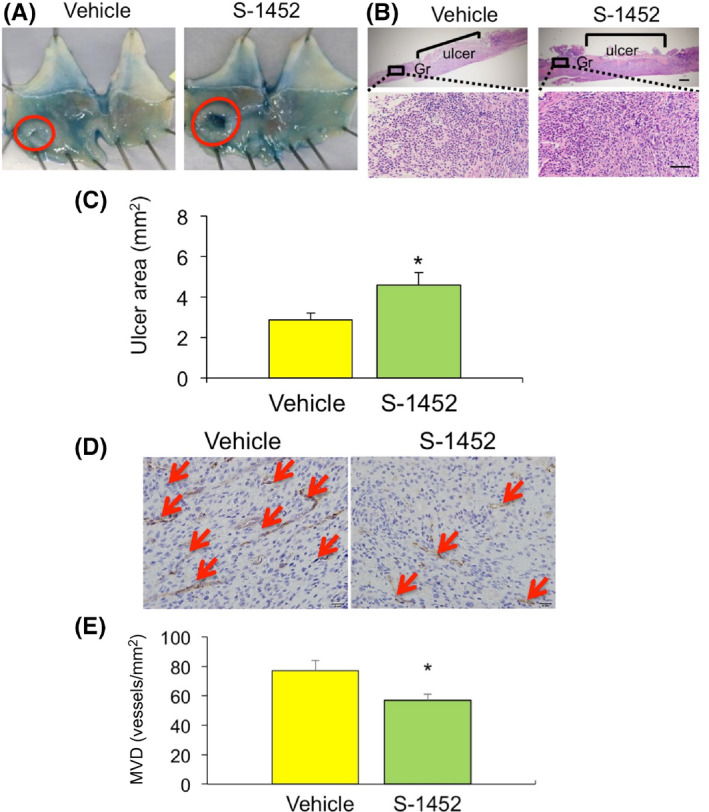
Effect of TP on healing of acid‐induced gastric ulcers. (A) Typical appearance of ulcers in S‐1452‐ and vehicle‐treated mice. (B) Haematoxylin–eosin staining in gastric ulcer lesions in S‐1452‐ and vehicle‐treated mice. Gr, granulation tissue, Bar = 200 μm (upper), Bar = 50 μm (lower) (B) Ulcer areas on day 7 in S‐1452‐ and vehicle‐treated mice. Error bars indicate the mean ± SD. *p < .05 versus vehicle by Student’s *t*‐test. (C) Representative immunohistochemical staining of CD31 in gastric ulcer lesions from S‐1452‐ and vehicle‐treated mice. Bar = 20 μm. (D) Microvessel density in ulcer granulation areas of S‐1452‐ and vehicle‐treated mice on day 7. Error bars indicate the mean ± SD. *p < .05 versus vehicle by Student’s *t*‐test

To assess the effects of TP on gastric ulcer healing, mice were treated with the selective TP antagonist S‐1452 or vehicle after ulcer induction. Ulcerated areas on day 7 were significantly larger (4.57 ± 0.63 mm^2^ versus 2.86 ± 0.34 mm^2^, *p* < .05, *n* = 5–6 per group; Figure 3A,B,C), and MVD around the ulcerated area significantly lower (56.9 ± 4.15 versus 77.0 ± 6.97 vessels/mm^2^, *p* < .05, *n* = 4 per group; Figure 3D,E) in mice treated with S‐1452 than with vehicle. These results indicated that TXA2‐TP signalling might be important for gastric ulcer healing.

### 3.3 Effect of endogenous TP signalling on gastric ulcer healing

**FIGURE 4:**
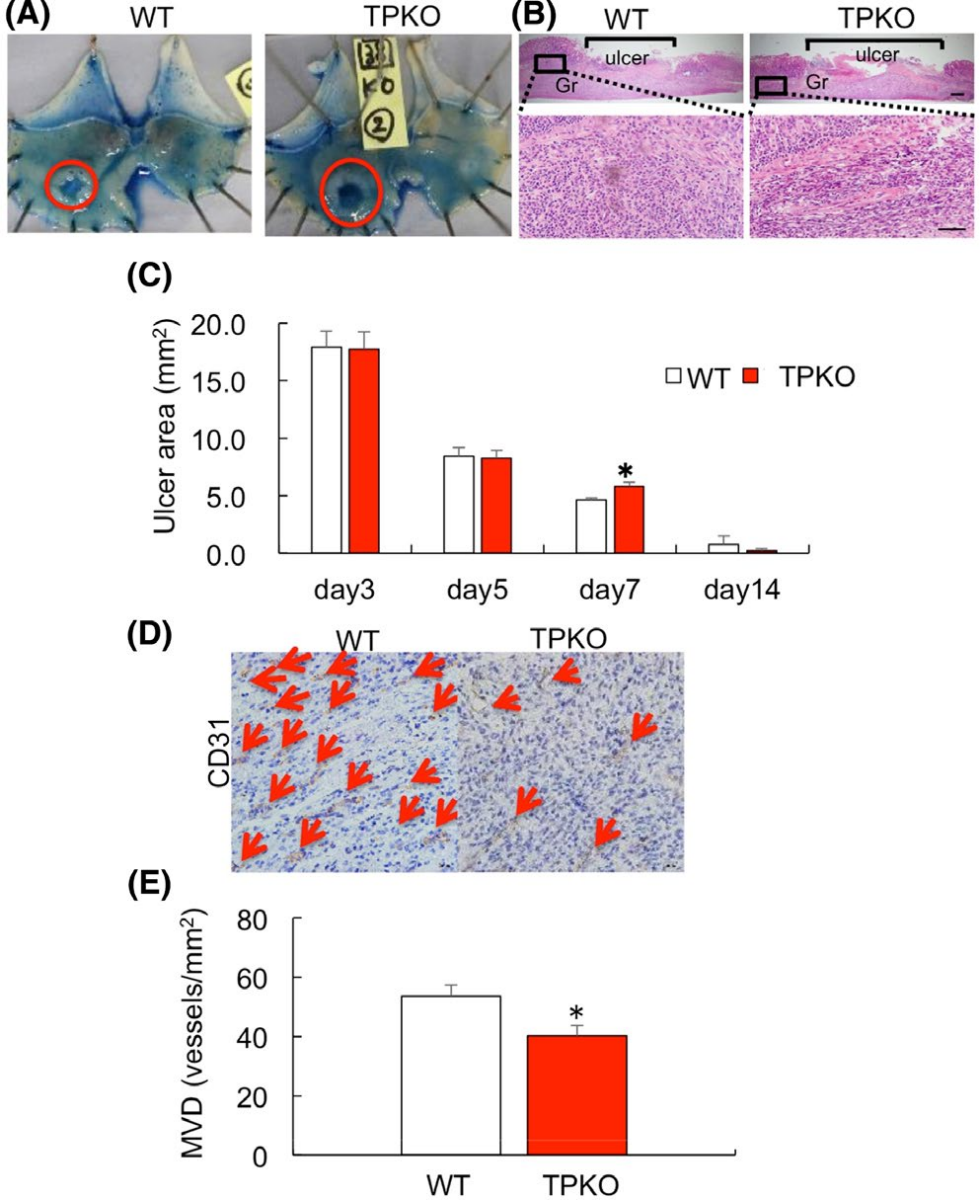
Effect of endogenous TP signalling on healing of acid‐induced gastric ulcers. (A) Typical appearance of ulcers in TXA2 receptor knockout (TPKO) and wild‐type (WT) mice. (B) Haematoxylin–eosin staining in gastric ulcer lesions in WT and TPKO mice. Gr, granulation tissue, Bar = 200 μm (upper), Bar = 50 μm (lower) (C) Time course of ulcer area changes in TPKO and WT mice. Error bars indicate the mean ± SD. *p < .05 versus WT by one‐way ANOVA. (D) Representative immunohistochemical staining of CD31 in gastric ulcer lesions of TPKO and WT mice. Bar = 20 μm. (E) Microvessel density in ulcer granulation areas of TPKO and WT mice on day 7. Error bars indicate the mean ± SD. *p < .05 versus vehicle by Student’s *t*‐test

The endogenous effects of TP receptor on gastric ulcer healing were determined by measuring the sizes of ulcerated areas and MVD in WT and TPKO mice. Ulcerated areas on day 7 were significantly larger (7.47 ± 1.06 mm^2^ versus 4.98 ± 0.36 mm^2^, *p* < .05, *n* = 20, 22 per group; Figure 4A,B,C), whereas MVD around gastric ulcer areas was significantly lower (40.2 ± 03.69 versus 53.5 ± 3.84 vessels/mm^2^, *p* < .05, *n* =8–12 mice per group; Figure 4D,E), in TPKO than in WT mice. These results indicated that endogenous TP signalling related to gastric ulcer healing by stimulating angiogenesis.

### 3.4 Involvement of platelet adhesion in gastric ulcer healing

**FIGURE 5:**
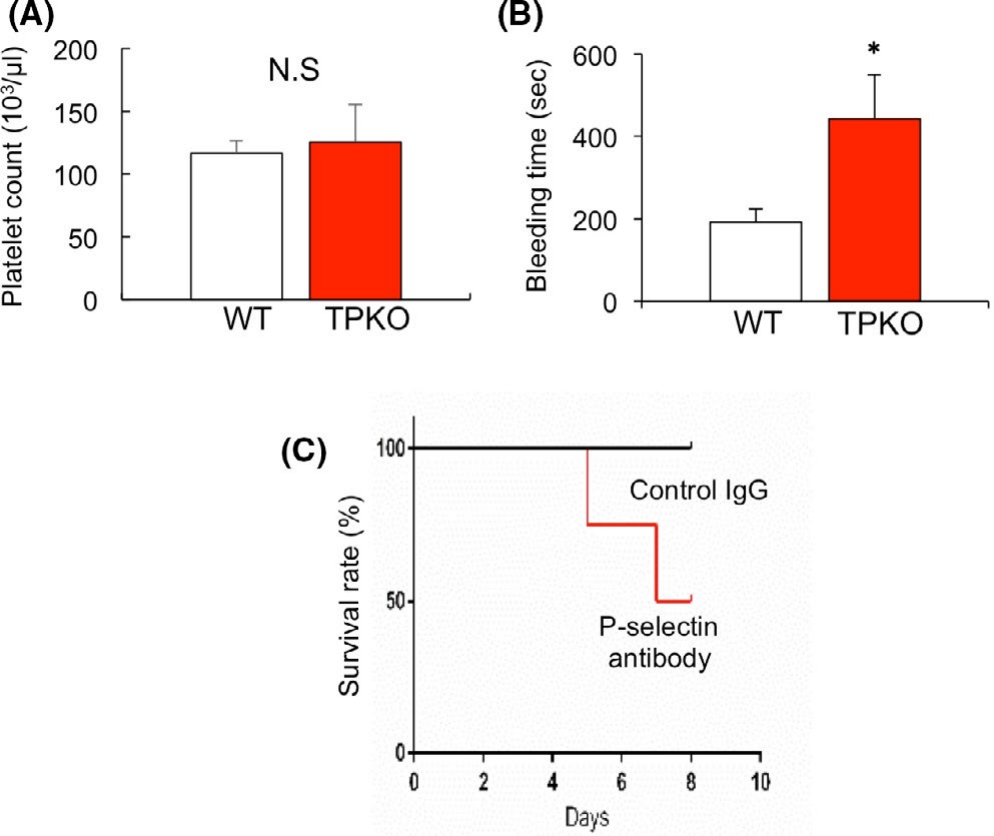
Effect of accumulation of activated P‐selectin^4^ platelets on gastric ulcer healing. (A) Platelet counts in TPKO and WT mice. Error bars indicate the mean ± SD. (B) Bleeding time in TPKO and WT mice. Error bars indicate the mean ±SD. *p < .05 versus vehicle by Student’s *t*‐test. (C) Kaplan–Meier analysis of survival after acid‐induced gastric ulcer in mice treated with anti‐P‐selectin antibody and control IgG

To determine whether altered platelet function in gastric ulcer affects ulcer healing, including platelet adhesion to microvessels, platelet counts and activation status were determined. Platelet counts did not differ significantly in TPKO and WT mice (125.5 ± 29.8 × 10^3^/μl versus 116.7 ± 10.1 × 10^3^/μl, *p* = .581, *n* = 5–9 mice per group; Figure 5A). By contrast, bleeding time was significantly longer in TPKO than in WT mice (442.5 ± 106.3 versus 191.0 ± 33.5 s, *p* < .05, *n* = 4–15 mice per group; Figure 5B). Activated platelets express P‐selectin.^18^ To determine whether platelets positive for activated P‐selectin are involved in gastric ulcer formation, mice were administered neutralizing antibody for P‐selectin following gastric ulcer induction. All mice treated with normal IgG survived, whereas only 40% of those treated with antibody to P‐selectin survived to day 7 (*p* < .05, *n* = 4–7 mice per group, Figure 5C). These results suggested that activated platelets were associated with gastric ulcer healing.

### 3.5 VEGF‐A and TGF‐β from platelets induce gastric ulcer healing

**FIGURE 6:**
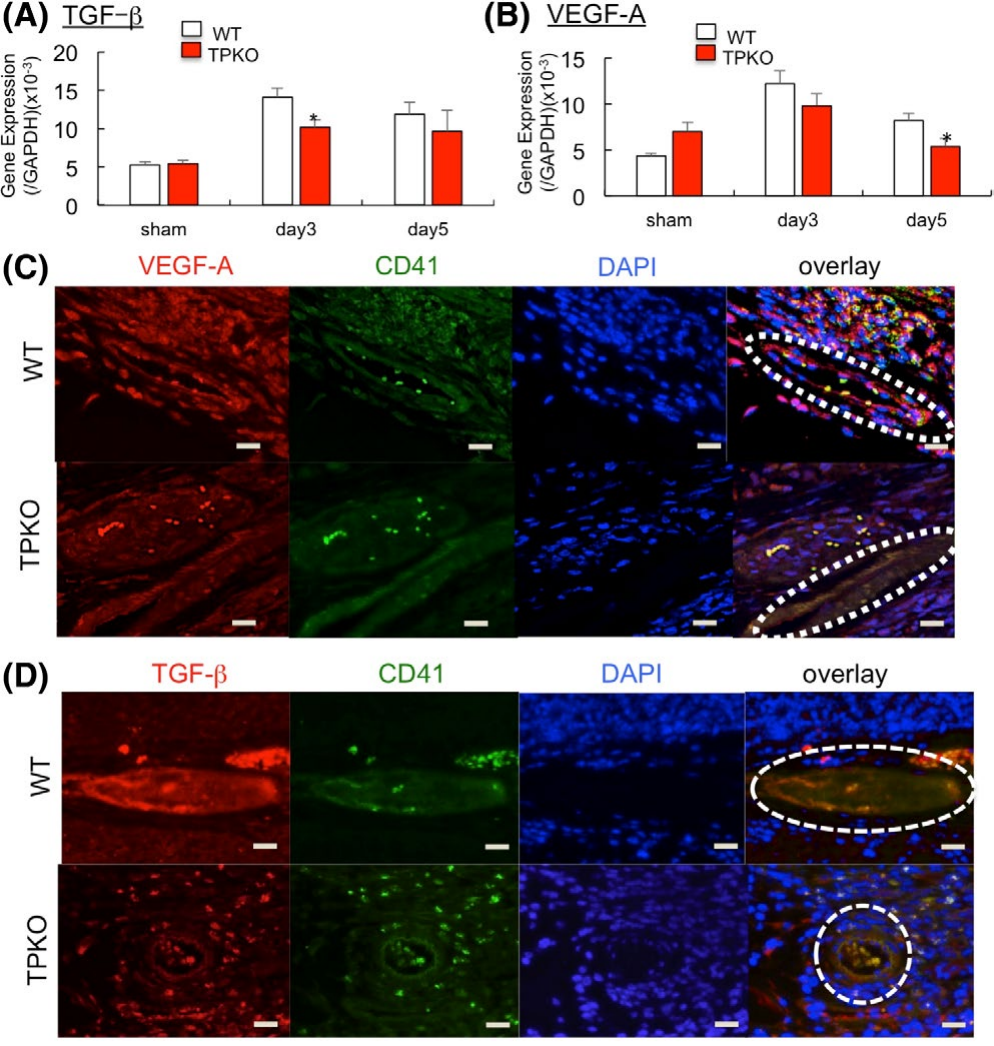
Effect of TXA2‐TP signalling on VEGF‐A and TGF‐β expression in gastric ulcers. Expression of TGF‐β mRNA (A) and VEGF‐A mRNA (B) expressions in ulcer granulation tissue from TPKO and WT mice on day 7. Error bars indicate the mean ± SD. *p < .05 versus WT by one‐way ANOVA. (C) Staining of TGF‐β^+^CD41^+^ platelets around endothelial cells in WT but not in TPKO mice. TGF‐β (red), CD41^+^ platelets (green), and DAPI (blue). Bar = 20 μm. Dotted circles indicate blood vessels. (D) Staining of VEGF‐A^+^CD41^+^ platelets around endothelial cells in WT but not in TPKO mice. VEGF‐A (red), CD41^+^ platelets (green), and DAPI (blue). Bar = 20 μm. Dotted circles indicate blood vessels

Because gastric ulcer healing was enhanced by angiogenesis and activated platelets secrete various angiogenesis stimulating factors, such as VEGF‐A and TGF‐β, the levels of VEGF‐A and TGF‐β mRNAs in ulcer granulation tissue were measured by real‐time PCR analysis. Expression of TGF‐β mRNA in ulcer granulation tissue was significantly lower in TPKO than in WT mice on day 3 (Figure 6A). Furthermore, expression of VEGF‐A was also lower in TPKO than in WT mice on day 5 (Figure 6B). These results suggested that TP signalling induced gastric ulcer healing through production of VEGF‐A and TGF‐β. Because angiogenesis is enhanced by platelet adhesion induced by TP signaling,^11^, ^19^ we utilized double fluorescence staining to determine whether platelet adhesion affects ulcer healing. Accumulation of CD41^+^ platelets on day 3 was observed in granulation tissue blood vessels of WT, but not TPKO, mice (Figure 6C), with the CD41^+^ platelets in WT but not TPKO mice being positive for TGF‐β on day 3 and VEGF‐A on day 5 (Figure 6D). These results indicated that TGF‐β and VEGF‐A released by platelets were involved in gastric ulcer healing and that this process was dependent on TP signalling.

### 3.6 Transplantation of BM cells from TPKO to WT mice impairs gastric ulcer healing

**FIGURE 7:**
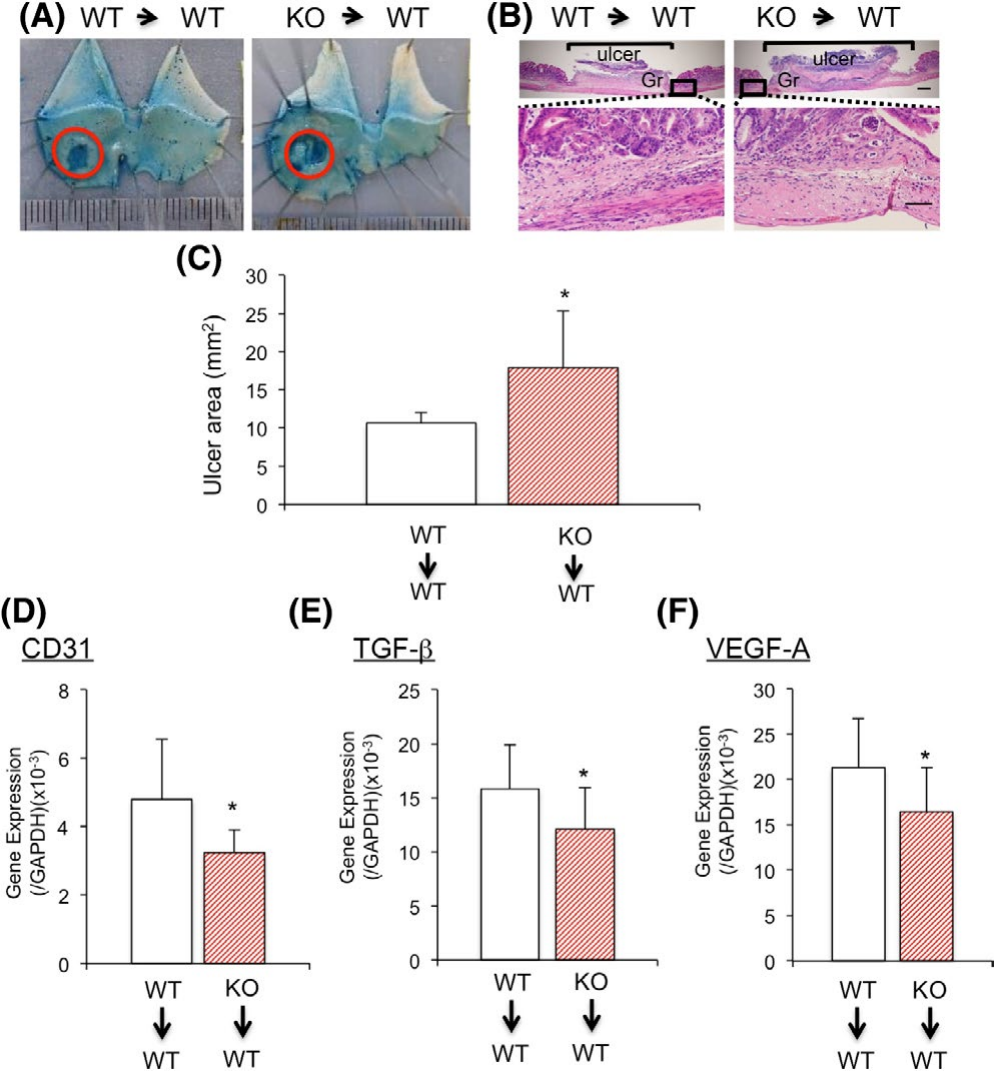
Effect of TP signalling in bone marrow (BM) on gastric ulcer healing. (A) Typical appearance of ulcers in TKKO‐BM→WT and WT‐BM→WT mice on day 7. (B) Haematoxylin–eosin staining in gastric ulcer lesions in TKKO‐BM→WT and WT‐BM→WT mice. Gr, granulation tissue, Bar = 200 μm (upper), Bar = 50 μm (lower). (C) Ulcer area changes in TKKO‐BM→WT and WT‐BM→WT mice on day 7. Error bars indicate the mean ±SD. *p < .05 versus vehicle by Student’s *t*‐test. Expression of CD31 mRNA (D), TGF‐6 mRNA (E) and VEGF‐A mRNA (F) in ulcer granulation tissue from TKKO‐BM→WT and WT‐BM→WT mice. Error bars indicate the mean ± SD. *p < .05 versus vehicle by Student’s *t* ‐test

BM‐derived hematopoietic cells have been shown to induce angiogenesis.^19^, ^20^ To determine whether BM‐derived platelets expressing TP contribute to gastric ulcer healing, we selectively inhibited TP signalling in BM cells by transplanting BM cells from TPKO and WT mice into WT mice. On day 7, the gastric ulcer area was significantly larger in TKKO‐BM→WT than in WT‐BM→WT mice (17.97 ± 3.31 versus 10.55 ± 0.54 mm^2^, *p* < .05, *n* = 5–7 mice per group; Figure 7A,B,C). Expression of CD31 was significantly lower in WT‐BM→WT than in TPKO‐BM→WT (Figure 7D). Furthermore, expression of TGF‐β and VEGF‐A in gastric ulcer tissue was lower in TPKO‐BM→WT than in WT‐BM→WT mice (Figure 7E, F). These results suggested that expression of TP by BM cells was involved in induction of gastric ulcer healing partly through VEGF‐A and TGF‐β.

## 4 DISCUSSION

The present study investigated the role of TXA_2_‐TP signalling in gastric ulcer healing. The results from mice lacking TP signalling demonstrated that TP signalling facilitates gastric ulcer healing and angiogenesis by enhancing VEGF‐A and TGF‐β secretion by platelet aggregates at areas of mucosal injury. Transplantation of BM cells isolated from TPKO mice into WT mice showed that BM‐derived platelets induced gastric ulcer healing and angiogenesis by enhancing VEGF‐A and TGF‐β expression dependent on TXA_2_‐TP signalling. TXA_2_‐TP signalling up‐regulated the expression of VEGF‐A and TGF‐β by BM‐derived platelet aggregates in areas of ulcer lesions, promoting angiogenesis and ulcer healing.

Gastric ulcers are caused by imbalances on the surface of gastric mucosa between mucosal defensive factors, including mucus, prostaglandins (PGs) and mucosal blood flow, and mucosal damaging factors, including acid and pepsin.^21^ Angiogenesis is an important process in all types of wound healing, including the healing of gastric ulcers. Angiogenesis is regulated by angiogenesis stimulating factors, such as VEGF, TGF‐β, fibroblast growth factor (FGF) and stromal cell derived factor‐1 (SDF‐1).^3^, ^4^

Platelets contain angiogenesis stimulating factors, including VEGF, TGF‐β, platelet factor, platelet derived growth factor (PDGF) and SDF‐1.^5^, ^6^ Platelets are involved in wound healing, recovery from ischaemia, tumour angiogenesis and metastasis formation.^9^, ^11^, ^19^ Transfusion of platelets or platelet‐rich plasma also induces angiogenesis.^22^, ^23^ Activated platelets that accumulate at sites of injury can deliver these factors and promote healing processes.

TXA_2_ synthase (TXS) is a downstream enzyme of arachidonic acid metabolism and catalyses the synthesis of TXA_2_, which stimulates platelet activation and aggregation. Fibroblasts overexpressing TXS induced recovery from ischaemia in a hind limb model.^19^ Expression of TXA_2_ and TP receptor were enhanced after induction of gastric ulcer formation.^24^ These results indicated that TXA_2_‐TP signalling was associated with gastric ulcer healing. We previously reported that OKY‐046 and S‐1452 suppressed neovessel formation and recovery from ischaemia in mice.^11^ Similarly, the present study showed that OKY‐046 and S‐1452 impaired neovessel formation and delayed gastric ulcer healing compared with vehicle in mice, further suggesting that TXA_2_‐TP signalling induces gastric ulcer healing. Furthermore, the current study showed that MVD in OKY‐046‐treated mice was lower than that in S‐1452‐treated mice. These results suggest that the anti‐angiogenic effect of TXS inhibition might be more potent than that of inactivation of TP receptor signalling.

Thromboxane prostanoid (TP) receptor is a receptor of TXA_2,_ which is coupled to the Gq family of heterotrimeric G proteins in vivo.^25^ Thromboxane prostanoid (TP) receptor localizes to cardiovascular, reproductive, immune, pulmonary and neurological tissues, among others.^26^, ^27^ TP has two isoforms, TP‐α and TP‐β, derived from alternate splicing of a single gene. These isoforms interact to coordinate platelet activation.^28^ To clarify how endogenous TP signalling affects gastric ulcer healing, we compared WT and TPKO mice, finding that ulcer healing and angiogenesis were impaired in TPKO mice. This finding strongly suggested that TXA_2_‐TP signalling plays an important role in ulcer healing.

Thromboxane A_2_ induces platelet activation, which, in turn, correlates with bleeding time.^29^ Despite WT and TPKO mice having similar platelet counts, bleeding time was longer in TPKO mice, with platelet activation being lower in TPKO than in WT mice. P‐selectin is expressed not only on platelets, especially activated platelets, but also on endothelial cells and is reported to be involved in tumour metastasis formation and recovery from ischaemic condition.^9^, ^11^, ^30^ Gastric ulcer formation and survival rate were reduced by injection of anti‐P‐selectin antibody. Because mice treated with anti‐P‐selectin antibody had a high mortality rate, we could not statistically determine its effect on gastric ulcer recovery. However, gastric ulcer recovery tended to be longer in mice treated with anti‐P‐selectin antibody than in mice treated with IgG, with the longer recovery likely due to bleeding and infection. These results suggested that platelet activation was a key regulator of gastric ulcer healing.

Platelets contain many pro‐angiogenic stimulating factors, including VEGF, TGF‐β and SDF‐1. In previous studies, using ischaemic hind limb and lung metastasis models, we showed that VEGF‐A and SDF‐1 expression were impaired in TPKO mice.^9^, ^11^ The present study also showed that the levels of TGF‐ β and VEGF‐A were lower in TPKO than in WT mice, but there was no difference in SDF‐1 expression (data not shown). Furthermore, immunofluorescence staining showed that staining for TGF‐β and VEGF‐A coincided with platelets (CD41) but was lower in TPKO than in WT mice. These results indicated that TXA_2_‐TP signalling induced aggregated platelets to secrete TGF‐β and VEGF‐A, which, in turn, contribute to ulcer healing.

Bone marrow (BM)‐derived cells are useful in recovering loss of function in damaged tissues. The levels of expressions of TGF‐β and VEGF‐A in gastric ulcer were lower in TKKO‐BM→WT than in WT‐BM→WT mice. Bone marrow‐derived cells promoted regeneration of the stomach in a rat model of ethanol‐induced ulcers.^31^ In addition, implantation of cultured BM‐derived cells has been shown to accelerate the healing of gastric ulcers.^32^

We also found that gastric ulcer healing was delayed in TKKO‐BM→WT mice, suggesting that BM‐derived platelets expressing TP induce ulcer healing and stimulate the secretion of VEGF‐A and TGF‐β1, stimulating localized angiogenesis and ultimately promoting ulcer healing. We previously showed that TP‐signalling‐dependent adhesion of activated platelets to endothelial cells in ischaemic muscle is a key regulator enhancing angiogenesis. These activated TP^+^ platelets released VEGF‐A and SDF‐1 and induced mobilization of VEGFR1^+^CXCR4^+^ cells from BM and their accumulation in ischaemic muscle and in gastric ulcer areas.^11^, ^14^ The present study did not determine the type of cells that accumulated in ulcer areas. Further experiments are needed to clarify this mechanism.

## 5 CONCLUSION

Thromboxane prostanoid (TP) receptor signalling in platelets promotes ulcer healing by enhancing the secretion of VEGF‐A and TGF‐β by platelets that had accumulated in ulcerated areas. Selective TP agonists may be attractive targets for promoting gastric ulcer healing.

## CONFLICT OF INTEREST

There are no conflicts of interest to declare.
